# Maternal immune activation in rats produces temporal perception impairments in adult offspring analogous to those observed in schizophrenia

**DOI:** 10.1371/journal.pone.0187719

**Published:** 2017-11-06

**Authors:** Ashley R. Deane, Jessica Millar, David K. Bilkey, Ryan D. Ward

**Affiliations:** Department of Psychology, University of Otago. Dunedin, Otago, New Zealand; Radboud University Medical Centre, NETHERLANDS

## Abstract

The neurophysiology underlying temporal perception significantly overlaps with areas of dysfunction identified in schizophrenia. Patients commonly exhibit distorted temporal perception, which likely contributes to functional impairments. Thus, study of temporal perception in animal models of the disease may help to understand both cognitive and neurobiological factors involved in functional impairments in patients. As maternal immune activation (MIA) has been shown to be a significant etiological risk factor in development of schizophrenia and other developmental psychiatric diseases, we tested interval timing in a rat model of MIA that has previously been shown to recapitulate several behavioural and neurophysiological impairments observed in the disease. Rats were tested on a temporal-bisection task, in which temporal duration stimuli were categorized as either “short” or “long” by responding to a corresponding lever. Data from this task were modeled to provide estimates of accuracy and sensitivity of temporal perception. Parameter estimates derived from the model fitting showed that MIA rats significantly overestimated the passage of time compared to controls. These results indicate that the MIA rat paradigm recapitulates timing distortions that are phenotypical of schizophrenia. These findings lend further support to the epidemiological validity of this MIA rat model, supporting its relevance for future research into the role of maternal immune activation in producing neurobiological and behavioural impairments in schizophrenia.

## Introduction

Accurate temporal perception is critical to normal daily functioning. Temporal perception requires the coordinated operation of a number of cognitive processes, including attention, working memory, and decision making [1], all of which are commonly found to be impaired in a variety of psychiatric and neurological disorders [2–4]. Therefore, the study of abnormal temporal-information processing stands to benefit not only our understanding of temporal processing mechanisms, but also our understanding of psychological and neurobiological mechanisms of psychopathology in clinical populations [5]. For example, the physiology and neurobiology involved in interval timing significantly overlaps with that implicated in schizophrenia [6,7]. Patients reliably demonstrate aberrant temporal perception [8], overestimating elapsed durations when they are asked to explicitly estimate interval length, and underestimating durations in tasks in which they are required to produce a temporal interval of a specified duration (e.g., finger tapping) [9,10]. Thus, patients show impaired accuracy of temporal perception, although the specific character of the impairment depends on the nature of the task. Notwithstanding the task-specific differences in accuracy impairments, when performance is considered across tasks, patients are reliably less precise in their estimates of time than controls [5,11]. This dysfunctional temporal perception may in turn contribute to other perceptual symptoms, including hallucinations and delusions, which are characteristic of schizophrenia [8,12].

Activation of the maternal immune system during gestation has been identified as a significant etiological risk factor in schizophrenia and other developmental psychiatric diseases, posited to predict 14–21% of all schizophrenia cases [13]. Following maternal exposure to viral pathogens during the first and early second trimester, there is increased incidence of later diagnosis of schizophrenia in offspring [13–15]. Upregulated pro-inflammatory cytokines in maternal serum have been associated with altered cellular communication [14], neuroanatomical abnormalities, and cognitive deficits [13], as well as a host of behavioural deficits in progeny, implicating maternal immune activation (MIA) as a causal factor in contributing to the unique constellation of symptoms seen in schizophrenia [16]. Thus, research into the role of MIA in the pathogenesis of schizophrenia may lead to improvements in diagnosis, prevention, and treatment.

The viral mimic polyriboinosinic-polyribocytidylic acid (Poly (I:C)) MIA model emulates a broad spectrum of histological, neuroanatomical, functional and behavioural deficits observed in schizophrenia [17–24]. As temporal-information processing deficits are phenotypical of schizophrenia, we assessed interval timing in the MIA rat model of schizophrenia using a temporal-bisection task, in which animals categorize the duration of elapsed cues as either “short” or “long” by responding on a corresponding response option. The resulting data can be analyzed to yield separable measures of accuracy and precision of temporal perception. This experimental paradigm is sensitive to temporal dysfunction in other animal models of schizophrenia [25]. Thus, we expected that the bisection procedure would be sensitive to any effects of MIA on temporal perception in rats. Given the broad correspondence in neurophysiological and behavioural disturbances seen in this MIA model and those observed in schizophrenia, we predicted impaired interval timing in adult offspring of MIA rats compared to controls.

## Methods

All procedures and experiments reported here were approved by the University of Otago Animal Ethics Committee.

### Subjects

Procedures for generating MIA rats have been detailed in previous research [26]. Briefly, 12 Sprague Dawley dams were mated at three months of age, with the following day defined as gestational day (GD) 1. On GD15, dams were administered isoflurane as anaesthesia and injected with Poly (I:C) (4.0mg/kg, i.v) dissolved in a saline solution. Control dams were injected with saline following an equivalent procedure. Four to six male offspring were kept per dam. They were weaned at three weeks of age, and subsequently housed in pairs within their litter groups.

Experimental participation began when offspring were three months of age. To facilitate motivation to participate in the experimental task, 20 rats (10 control, 10 MIA) were maintained at 85% of their free feeding weight during experiments. They were weighed daily and fed any supplemental chow after the day’s testing session. Water was available *ad libitum*. Rats were maintained on a 12 hour light/dark cycle (6am to 6pm). All experimental testing was conducted during the light phase. No more than two offspring per litter were used in experiments.

### Apparatus

#### Operant chambers

The operant chambers (Med-Associates, St. Albans, VT; model ENV-008), with internal dimensions 30.5 cm x 24.1 cm x 21.0 cm, were located in a light and sound attenuating cabinet equipped with an exhaust fan. This fan provided 72dB of background white noise. The floor was comprised of metal rods spaced 0.87cms apart. Each chamber was equipped with a food hopper in a central location on one chamber wall to which food pellets (used as the reward in this experiment) could be delivered. Chambers were also equipped with two retractable levers, located on either side of the food hopper. An infrared photocell detector was used to record head entries into the hopper. A house light provided chamber illumination. An audio speaker was positioned 8.5 cm from the floor on the wall opposite the food hopper. All output programming and data recording was completed using Med-PC IV.

### Procedure

Temporal perception was assessed using a temporal-bisection procedure. In the temporal-bisection procedure, originally used by Church and Deluty [27], animals are trained to discriminate anchor cues by being rewarded for pressing one lever following short (e.g, 2s) cues and the other following long (e.g. 8s) cues. Following initial training, unreinforced intermediate duration probes are presented on half of the trials, which are categorized by the animals as either short or long by responding on either the lever associated with the 2s or 8s sample. Plotting the proportion of long responses as a function of sample duration generally produces an increasing sigmoidal function [9] which can then be analyzed further to obtain quantitative measures of several separable aspects of temporal perception (see Data Analysis section).

Experimental sessions occurred once per day, 5–7 days per week. Training proceeded in several phases. The criterion to proceed to the next choice training phase was defined as 80% correct choice. During initial training, all trials were separated by a variable inter-trial interval with an average of 30s.

#### Training phases

Pellet training. Rats were trained to retrieve pellets from the hopper over three sessions of 60 trials each. Pellets were delivered on average every 30 s. By the end of the three sessions of training, rats were retrieving at least 55 of 60 pellets.

Lever press training. On each trial, either the right or left lever was presented and a response within 10 s resulted in delivery of a reward. Failure to respond within 10 s resulted in lever retraction and no reward. Rats completed three sessions of 60 trials, by the end of which animals were lever pressing on more than 80% of trials.

Single cue/single lever training. Rats were trained to associate a tone stimulus of a particular duration with a specific lever. Each trial consisted of presentation of a temporal-duration cue, followed by insertion of the corresponding lever. A response on the lever was rewarded. Lever assignment was counterbalanced for the two anchor cues (2 and 8 s). Rats received two sessions of 70 trials with each cue and lever presented alone, followed by two sessions in which both cues and levers were presented an equal number of times. For this and all subsequent phases, the inter-trial interval was increased to an average of 45 s.

50% choice response trials. Fifty percent of the trials in a session involved presenting an anchor cue followed by the extension of the associated lever. The remaining fifty percent of trials were choice response trials which involved the extension of both levers following presentation of either of the anchor cues. Rats would receive rewards for a response on the lever associated with the presented sample duration. Correction trials followed incorrect responses. During correction, the incorrect trial was repeated again, with the same sample duration presented until the rat made the correct choice. Two sessions of 70 trials were completed.

100% choice response trials. In this stage, all of the trials required the rats to make a choice response. This stage lasted for two sessions.

100% choice training without correction. All trials required the rats to make a choice response. This phase lasted for nine sessions. The correction was turned off during this phase. In order to progress to the experimental phase, rats had to correctly distinguish the 2 & 8s cues on 80% of trials per session, for at least 4 consecutive sessions.

Intermediate duration testing. Rats performed the temporal-bisection task with 100% choice trials, but intermediate cue durations (2.6, 3.2, 4, 5, 6.4s) were included [25]. These were presented with anchor cues (2 and 8s) in randomized order. Rats received rewards for correct responses to anchor cues only. Each sample duration was presented 10 times per session. Rats completed 8 sessions.

### Data analysis

To assess temporal-bisection performance, we analyzed the proportion of responses made to the “long” lever following presentation of the 2 and 8s cues during initial training. For the intermediate testing phases we analyzed the proportion of long responses made following presentation of all cue durations. In addition, we also analyzed head entry and choice latency. Results were analyzed with repeated measures ANOVAs with appropriate factors.

#### Curve fitting

To determine the specific aspects of temporal discrimination that differed between MIA and control rats, the proportion-long data were averaged across the final five sessions of intermediate sample testing and fit by a cumulative normal function with four parameters. The function fitted to the data was

$$f\left(t\right)=a+\frac{b}{\sqrt{2\pi \sigma }}\;\left[\exp-\left(\frac{{(t-\mu )}^{2}}{2\sigma^{2}}\right)\right]$$ Eq 1

where *f*(t) is the proportion of long responses at a given sample duration (t), *a* is the lower asymptote of the function, b is the range of the function (upper–lower asymptote), μ the mean, and σ the standard deviation [28]. The parameters of the function give measures of three separable aspects of temporal-discrimination performance. The range of the function reflects the degree to which choice behaviour is under control of the temporal-sample stimuli. This has been interpreted as an indication of the degree to which the animals attend to the task [28]. The mean is the point on the function where the proportion of long responses is .50. This is the point of subjective equality (PSE; accuracy of timing) and quantifies lateral shifts in the function. Shifts to the left and right have been interpreted as indicating over and underestimation of time, respectively [29]. The standard deviation is the slope of the function. It is a measure of the sensitivity to the differences between the short and long temporal durations, or the precision of timing.

We fit the model to the average proportion long data from each rat using the Solver tool of Microsoft Excel. The regression proceeds for successive iterations until it maximizes the variance accounted for by the equation. The fits of the model to the data from individual rats were excellent, accounting for >95% of the variance in the individual proportion long functions. Derived parameters were compared using a between subjects t-test.

## Results

One control animal was excluded from data analysis due to lever bias.

**Fig 1:**
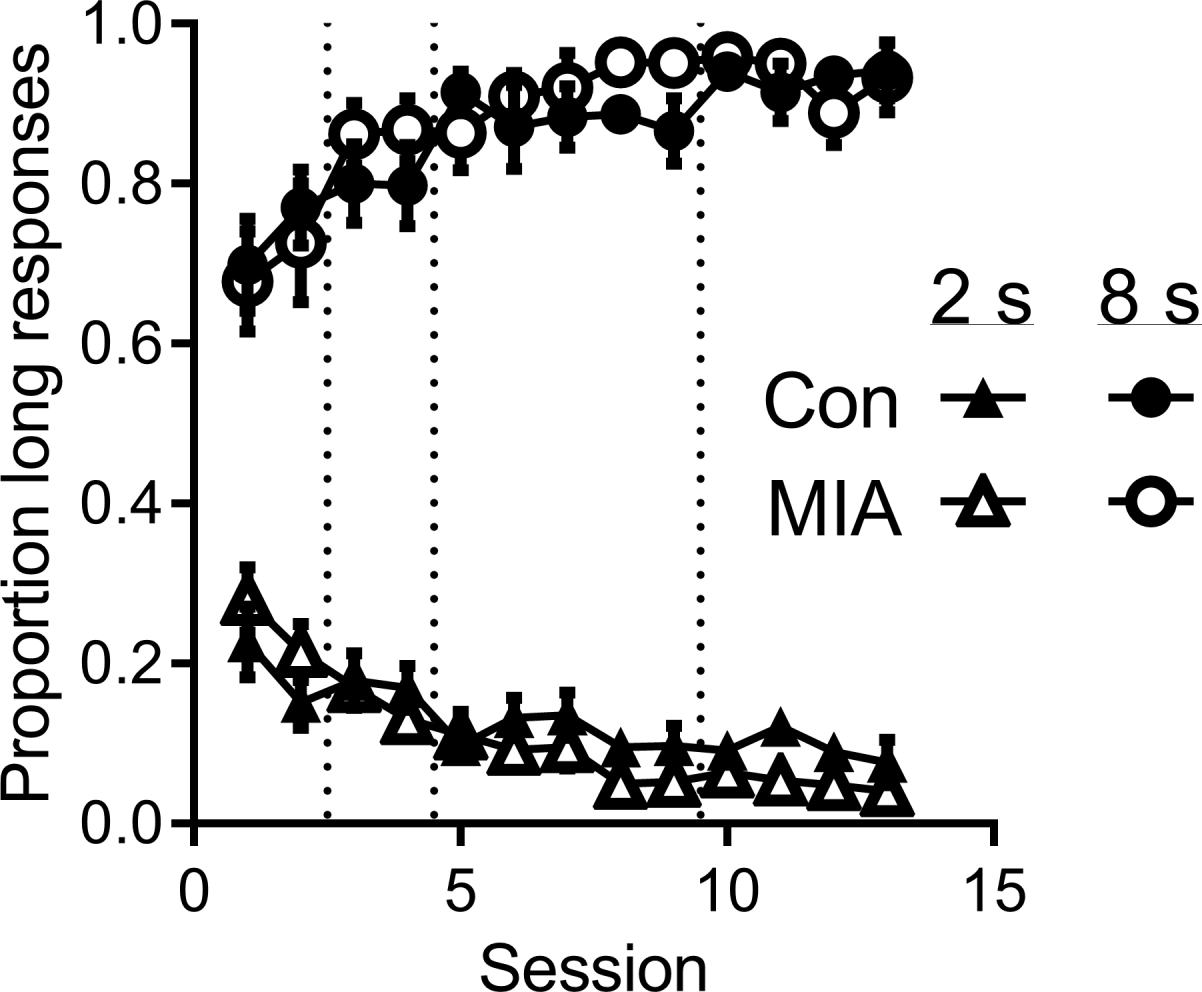
Mean proportion of responses made by MIA (N = 10) and control (N = 9) rats to the lever corresponding to the “long” cue duration during both 2 and 8 second anchor cue trials across training phases (50% choice, 100% choice with correction, 100% choice without correction (first and second epochs); indicated by dashed lines). Error bars indicate standard error of the mean.

Fig 1 shows the proportion of responses to the “long” lever following 2 and 8 s sample durations as a function of training phase for MIA and control rats. Three aspects of the data in the figure are readily apparent. First, rats responded differently to the different durations. The proportion of long responses to the 2 s duration was low, whereas the proportion of long responses to the 8 s duration was high. This difference was apparent from the outset of training, and persisted throughout the training phases. Second, rats became more accurate in their categorization of the 2 and 8 s cue durations as training progressed. Third, there was no difference in discrimination of the two temporal intervals between MIA and control rats.

A repeated measures ANOVA was used to analyze the training data with respect to training phase, cue duration, and group. Data was averaged across the two training sessions for both the 50% and 100% training phases, while averages were taken over the first and last halves of 100% no correction training. These analyses revealed a main effect of cue duration (*F*_(1,17)_ = 989.27, *p* < 0.001), indicating that the animals were able to differentiate between the 2 and 8 s cues. While there was no significant effect of training phase (*F*_(3,51)_ = 0.734, *p* = 0.537), there was a significant cue duration x training phase interaction (*F*_(3,51)_ = 21.85, *p* < 0.0001), indicating improved discrimination of the two durations as training progressed. No main effect of group was found, indicating that there were no significant differences between the training data of MIA and control rats (*F*_(1,17)_ = 0.224, *p* = 0.642). There was also no interaction between cue duration and group (*F*_(1,17)_ = 0.00, *p* = 1.00), and no interaction between group and training phase (*F*_(3,51)_ = 0.328, *p* = 0.805). The three way interaction between group, training phase and cue duration was also not significant (*F*_(3,51)_ = 1.179, *p* = 0.327).

In order to locate the source of the significant interactions in the training data, separate ANOVAs on data from the 2 and 8 s cue durations were conducted over the four key stages in training. The analysis for the 2 s cue duration found a significant effect of training phase (*F*_(3,51)_ = 20.39, *p* < 0.0001), but no effect of group (F_(1,17)_ = 0.317, p = 0.581), and no training phase x group interaction (*F*_(3,51)_ = 2.498, *p* = 0.07). Likewise, the analysis of the 8 s cue found a significant effect of training phase (*F*_(3,51)_ = 7.453, *p* < 0.0001), but no effect of group (*F*_(1,17)_ = 0.049, *p* = 0.828), or group x training phase interaction (*F*_(3,51)_ = 0.327, *p* = 0.806).

In order to assess whether there were differences in acquisition across groups within training phases, we conducted separate analyses of the data from each training phase. For these analyses we compared the data from the first session of each of the first training phases (50% choice and 100% choice with correction) to those from the second session. For the final phase (100% choice with no correction) we separated the data into the first five and the last four sessions (referred to below as epochs; as in Fig 1). These analyses in all cases found only significant effects of duration (all Fs > 200) and in the case of the final training phase found a significant training epoch x duration interaction (p = .018), evidenced by the continued improvement on 2 s trials across the final phase of training.

Overall, these data indicate that both control and MIA rats learned the temporal discrimination. Furthermore, there was no evidence of any differences in acquisition during any phase of acquisition. Finally, performance was equivalent prior to the intermediate testing phase.

Additionally, average choice response latency was recorded across the training phases. Due to a recording error, latency data was only available for 15 rats (8 MIA, 7 control). Choice latency decreased as training progressed, indicating that rats became more efficient as their performance improved. The average latency (standard error in parentheses) for control and MIA rats decreased from 1.79 (0.14) and 1.55 s (0.14), respectively, during the first training phase to 0.74 (0.06) and 0.50 (0.02), respectively, during the last training phase. Furthermore, MIA rats responded consistently faster relative to controls, over all stages of training (control = 1.04 (0.05), MIA = 0.83 (0.05)). A repeated measures ANOVA showed that there was a significant effect of training phase (*F*_(3,39)_ = 86.059, *p* < 0.001). There was a significant main effect of group (*F*_(1,13)_ = 7.752, *p* = 0.015), but no significant training phase by group interaction (*F*_(3,39)_ = 0.79, *p* = 0.971).

**Fig 2:**
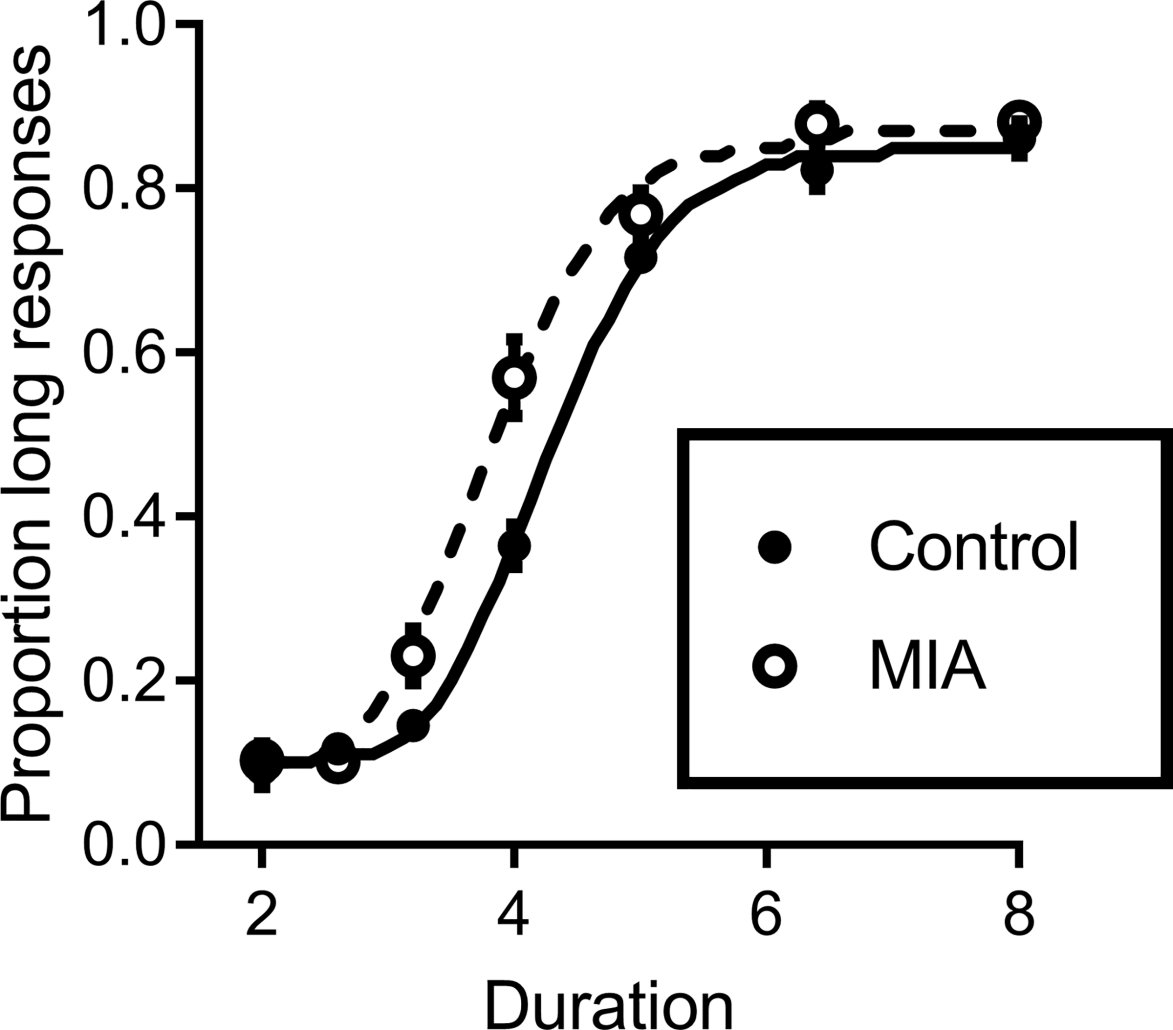
Mean proportion of responses to the lever corresponding to the “long” anchor cue as a function of sample duration for control and MIA rats during the intermediate sample testing phase. The lines through the data are the best fitting form of Eq 1 to the average proportion-long response data across rats from each group (parameter estimates are based on fits of the model to individual rats). Please note that error bars are subsumed by the data points in some cases.

Fig 2 shows the proportion of long responses made by MIA and control rats during testing with intermediate cue durations. The proportion of long responses increased with increasing cue duration for both MIA and control rats. Additionally, compared to the control animals, the proportion-long function of the MIA rats was shifted to the left.

The proportion-long data were analyzed using a repeated measures ANOVA with cue duration as a within subjects variable and group (MIA vs. control) as a between subjects variable. The ANOVA found a significant effect of cue duration (*F*_(6,102)_ = 562.137, *p* < 0.001), evidenced by the increasing proportion of long responses with increasing cue duration. There was a main effect of group (*F*_(1,17)_ = 6.43, *p* = 0.021) and a significant group x duration interaction (*F*_(6,102)_ = 6.076, *p* < 0.001), indicating that although the proportion-long response functions increased for both MIA and control rats, the nature of the increase differed across groups.

We also analyzed the latency to make a choice response and the latency to retrieve the reward. Due to a recording error, data for these analyses were available for only 15 rats (8 MIA, 7 control). The results showed that the average choice response latency for MIA rats (in seconds, with standard error in parenthesis) was 0.60 (0.06), while that for controls was 0.80 (0.09). This difference neared statistical significance (t_(13)_ = 2.123, p = 0.054). We also assessed whether there were any differences in latency across the different durations. An ANOVA showed no significant effect of duration (effect of duration; *F*_(6,78)_ = 0.634, *p* = 0.70), or group (effect of group; *F*_(1,13)_ = 3.96, *p* = 0.07), and no significant interaction. In terms of head-entry latency, MIA rats were significantly faster (0.13 (0.02)) than controls (0.21 (0.02)); t_(13)_ = 2.809, p = 0.014).

**Fig 3:**
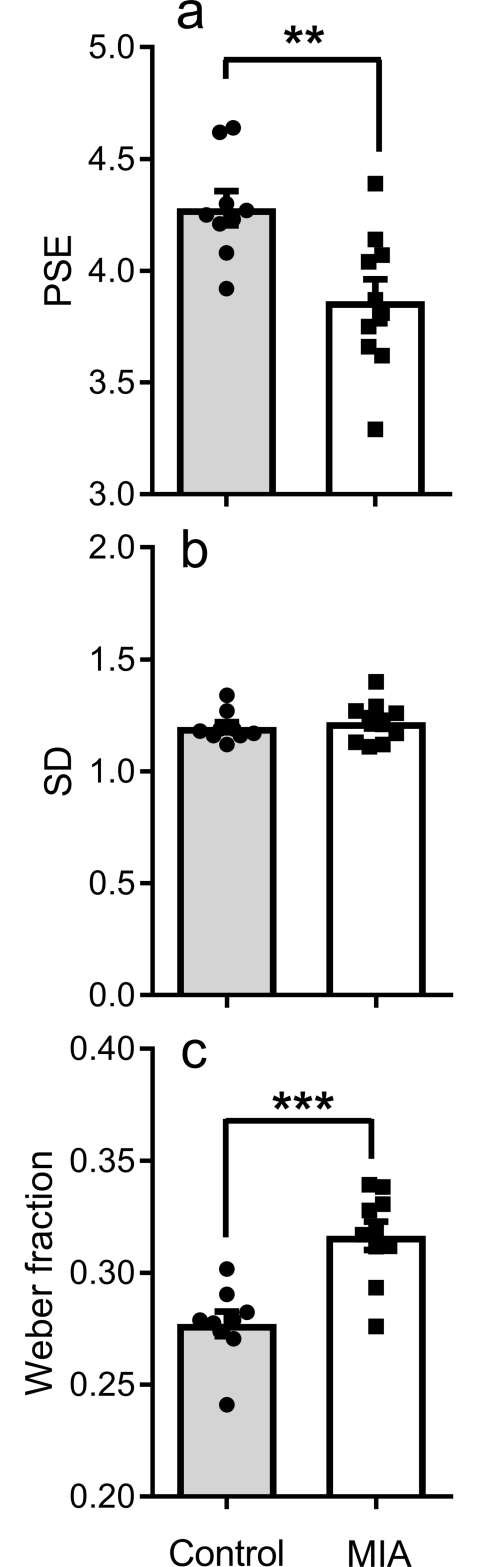
Mean best fitting parameters derived from the fitting of the theoretical model to the proportion long data from control and MIA rats. Panel a) shows the point of subjective equality, panel b) shows estimates of the standard deviation, panel c) shows the weber fraction. Data points indicate data from individual rats. Other details as in Fig 1.

Fig 3 shows the parameter estimates obtained from fitting the model to the data from the intermediate testing phase. This analysis was undertaken to isolate the specific aspects of timing in MIA and control rats. As is clear from the data in Fig 2, there was no difference in the range of the function between control and MIA rats (MIA = 0.79, control = 0.743, (t_(17)_ = 1.238, p = 0.232), indicating that behaviour of both groups was under control of the temporal-sample stimuli. The top panel of Fig 3 shows the point of subjective equality (PSE), or the bisection point, the measure of timing accuracy, for the respective groups. The PSE was lower for MIA rats than controls (corresponding to the leftward shift in the data). An unpaired t test found a significant difference between PSE values of MIA rats and controls (t_(17)_ = 3.28, p = 0.004), indicating that MIA rats overestimated the amount of elapsed time relative to controls.

The middle panel shows the estimates of the standard deviation of the function (timing precision). There were no differences in the precision of temporal-information processing between control and MIA rats. This was confirmed by an unpaired t test on the parameter estimates (t_(17)_ = 0.60, p = 0.56). Finally, there were no significant differences in parameter estimates of the lower asymptote (MIA = 0.091, control = 0.104, (t_(17)_ = -0.532, p = 0.602).

As a measure of sensitivity, we calculated the weber fraction, which is the PSE divided by the standard deviation of the function. The bottom panel shows that the weber fraction was greater for MIA rats than controls. This difference was significant (t(17) = 4.65, p = 0.0002). Together, these data show that while the behaviour of both groups was under the control of the temporal durations (and sensitive to the differences between short and long cues), MIA rats overestimated the elapsed time and were less sensitive to time relative to controls.

## Discussion

The aim of this study was to identify and subsequently characterize any temporal-perception deficits produced by MIA in a rat model of schizophrenia and other developmental psychiatric diseases. To our knowledge, this is the first assessment of interval timing in the MIA model. Analysis of the data from the training phase found no difference in acquisition of the temporal bisection task between MIA and control rats. Upon exposure to the intermediate durations during the testing phase, both groups of animals were shown to be sensitive to the differences between the short and long cues, as evidenced by the increasing proportion of long choices with increasing cue duration. However, the proportion long function of MIA rats was shifted leftward, indicating overestimation of the passage of time. These results were confirmed by quantitative analysis of the proportion long choice data, which indicated significant differences between MIA and control rats in the estimates of timing accuracy. Finally, the weber fractions for MIA rats were significantly greater than for controls, indicating decreased overall sensitivity to time.

Our results are strikingly similar to those from patients diagnosed with schizophrenia. Patients overestimate when asked to evaluate the duration of elapsed time [4,11]. They also display deficits in a number of cognitive processes that are integral to accurate temporal-information processing, such as working memory, attention, and decision making [1,30]. The present results, when considered in conjunction with the other deficits in cognitive function identified following the MIA intervention [17–21], add support to the notion that the MIA rodent is a valid model of aspects of the symptomology seen in human manifestations of schizophrenia.

There are several aspects of temporal perception that, if impaired, could lead to overestimation of time. According to scalar expectancy theory (SET), the dominant model of timing [31,32], in order for accurate timing to occur, the occurrence of a significant event (a time marker) must capture attention, thereby initiating the accumulation of temporal “pulses” (via a clock mechanism) into short-term memory. Once a biologically significant event, such as the delivery of food to a hungry animal, occurs, the number of pulses in the accumulator is then transferred into long-term memory. On subsequent trials, the current amount of pulses in the accumulator is compared with a value from long-term memory and if the difference is smaller than a criterion threshold, an appropriate behavioural response occurs.

Shifts of the sort seen in the present study are typically thought to indicate effects on the clock/accumulator phase of temporal information processing [33–37]. These clock-speed changes are most readily observed when animals are trained under one steady state condition and tested under another, such as administration of a drug. The faster clock under drug is thought to lead to more pulses in the accumulator at the end of the to-be-timed interval, and therefore to lead to overestimation of time [29,36]. However, given that time perception depends on the relative, not the absolute difference between temporal stimuli [31], and given the fact that MIA rats were trained and tested in the same state, a basal increase in clock-speed would only add a constant to the relative durations of the temporal stimuli. Thus, the ratio between the “short” and “long” durations would be the same for MIA as for controls and would therefore not produce the shifts in the functions we observed.

Given the intact discrimination of the 2 and 8 second anchor cues during training and testing, it seems unlikely that the issue is related to working or long-term memory impairments. This is because, like the clock-speed interpretation, problems of encoding, storage, or retrieval would likely have similar effects for short and long duration stimuli. Adding or subtracting a constant would not change the ratio between the short and long sample durations, so any accounts based on memory would have to posit differential effects on short vs long sample stimuli. Such effects are not easily explained by current models of timing.

One possible mechanism could be differences in attention between MIA and control rats. According to SET, attention controls the switch which gates pulses from the pacemaker into the accumulator. In order for an attentional effect to produce overestimation according to the model, MIA rats would have to be quicker to attend and to pay more attention to the temporal-sample stimuli, thus closing the switch and accumulating pulses faster than controls. Here once again, however we encounter the same problem as above, and would have to posit differential effects on short vs long samples. In addition, in the bisection procedure, manipulations that purportedly impact attention have been shown to flatten the psychophysical function, not shift it [28,38–40].

Another possible contributing factor is differences in motivation between MIA and controls. The latency data suggest that MIA animals were perhaps more motivated than controls, at least in retrieving the reward. Although motivation (or arousal) is incorporated into scalar expectancy theory, it does not impact timing according to the model. It is clear, however, that motivational variables do, in fact, impact timing, and they have been more explicitly incorporated into other models of timing [41,42]. Motivational manipulations are thought to predominantly impact the clock phase of temporal-information processing (though it is possible that they could impact or interact with other stages of temporal-information processing; see [5] for discussion), such that increased motivation increases the rate of the pacemaker [42]. Manipulations that target motivation can have varied, but often transient, effects depending on the timing procedure employed, shifting and/or flattening the function relating responding to temporal duration [28,38,39,43–48]. Much less work has been conducted on the effects of basal differences in motivation. Thus, while it is possible that some combination of attention, memory, or clock speed could interact with motivational variables in producing the present results, an interpretation based on current models of timing is not readily apparent.

One possible way that a basal difference in motivation could impact timing is by changing the decision threshold. There are at least two ways that this could occur. Differences in motivation could change the mean threshold for categorizing a presented sample as short or long, or it could change the variability of the threshold from trial to trial. Changes of the mean threshold would lead to shifts in the PSE, while changes in the variability of the threshold would lead to changes in the slopes of the bisection function [49]. Moreover, any changes in the decision threshold would be long-lasting. Thus, the observed shifts in the function in the present study are consistent with a change in the mean decision threshold in MIA rats.

Most data from timing procedures has found that as the target interval increases, the slope of the function relating timing to signal duration decreases, indicating increased variability (decreased sensitivity) of time perception. This is thought to be a function of endemic variability in the timing system increasing with increasing signal duration. Nevertheless, this increase in timing variability is proportional to the signal duration, such that the weber fraction (ratio of the PSE to the standard deviation) generally remains constant across a wide range of signal durations (the scalar property of timing; [31]). The fact that the weber fraction was greater in MIA rats indicates that the slope of the psychophysical function did not increase proportionally to the decrease in the PSE in these animals; that is, MIA animals would be predicted to become more sensitive to the differences between the short and long durations than controls due to their significantly lower PSE. This increase in sensitivity was not observed. Further manipulation of temporal parameters is necessary in order to find specific mechanisms underlying the differential temporal sensitivity between MIA and control rats seen here.

In conclusion, the present data represent the first attempt to characterize temporal-information processing in the MIA model of schizophrenia risk. Our results show that MIA is sufficient to produce deficits in temporal perception that are strikingly similar to those manifested by patients with schizophrenia. These results will serve as a foundation from which to undertake future explorations of the cognitive and neurophysiological mechanisms underlying these impairments and will aid in further elucidation of the role of MIA in the development and manifestation of timing impairments and their connection to specific symptom clusters in schizophrenia and other neurodevelopmental diseases.

## Supporting information

S1 Dataset Proportion of responses to the lever corresponding to the “long” duration stimulus for MIA and control rats during the intermediate phase. (XLSX)
